# Supplementary material for: Prevalence and associated factors with long COVID in the Brazilian population: The role of health-related behaviors and sociodemographic characteristics
Source: PLoS One. 2026 Jan 2;21(1):e0339612. doi: 10.1371/journal.pone.0339612 (PMC12758746; doi:10.1371/journal.pone.0339612)
Supplement: S1 File — (DOCX) [file pone.0339612.s001.docx]

STROBE Statement—Checklist of items that should be included in reports of ***cross-sectional studies***

|  | Item No | Recommendation | Relevant text from manuscript |
| --- | --- | --- | --- |
| **Title and abstract** | 1 | (*a*) Indicate the study’s design with a commonly used term in the title or the abstract | Page 1 Prevalence and associated factors with long COVID in the Brazilian population |
|  |  | (*b*) Provide in the abstract an informative and balanced summary of what was done and what was found | Page 2-3 The disease caused by the 2019 coronavirus (COVID-19) has resulted in unprecedented morbidity and mortality worldwide, with many individuals experiencing persistent symptoms and a decline in quality of life after infection. This study aims to analyze the prevalence and associated factors of long COVID in the Brazilian population, focusing on disease severity and immunization status.This observational, cross-sectional web survey employed a quantitative approach to analyze data from 4,231 participants, focusing on the prevalence and associated factors of long COVID. Descriptive statistics were performed, then a Fisher's exact test was conducted to analyze the outcome variable in relation to other qualitative variables in the database. Following this, a logistic regression model was adjusted to identify which variables were associated with the outcome of interest. To determine the best model, a stepwise selection method was employed, and the model's performance was assessed utilizing a Receiver Operating Characteristic Curve (ROC). The findings revealed a long COVID prevalence of 56.4% (2,386 cases), with men having a 36.46%(OR = 1,3646 ; CI = [1,1737 ; 1,5865]) higher chance of developing long COVID compared to women. A prior diagnosis before vaccination increased the risk by 22.30%(OR = 1,2230 ; CI = [1,0587 ; 1,4126]). Additionally, the use of sedatives and alcohol was linked to increased risks of 24.50%(OR = 1,2450 ; CI = [1,0782 ; 1,4378]) and 34.95%(OR = 1,3495 ; CI = [1,0272 ; 1,7569]), respectively. Beneficiaries of social programs faced a 47.29%(OR = 1,4729 ; CI = [1,2726 ; 1,7099]) higher risk, while individuals with comorbidities had a 33.47% (OR =1,3347 ; CI = [1,2004 ; 1,4866]) increase in risk. Hospitalization significantly raised the likelihood of prolonged symptoms by 331.92% (OR = 4,3192 ; CI = [2,5377 ; 7,8782]). Overall, various factors, including sedative and alcohol use, were associated with higher long COVID risk, whereas vaccination showed a positive impact, suggesting that association models can help healthcare professionals identify high-risk patients and tailor care effectively. |
| Introduction | | |  |
| Background/rationale | 2 | Explain the scientific background and rationale for the investigation being reported | Page 3-4 The disease caused by the 2019 coronavirus (COVID-19) has resulted in unprecedented morbidity and mortality worldwide. In Brazil, as of October 2024, the number of individuals affected by COVID-19 reached 38,953,513, with 713,626 deaths and over 38 million recoveries (1). Additionally, data from the Ministry of Health indicate that the number of cases and deaths has impacted different regions of the country variably, with the Central-West region showing the highest incidence of cases at 28,037.8 per 100,000 inhabitants and the highest death rate at 412.4 per 100,000 inhabitants as of October 2024, revealing the significant socio-spatial inequalities that characterize the country (2).  Regarding the impact of COVID-19 among recovered individuals, scientific and clinical evidence is evolving concerning the subacute and long-term effects of this disease, which can affect multiple systems and organs. Reports suggest effects of SARS-CoV-2 infection, with the main symptoms being fatigue, muscle pain, cough, shortness of breath, chest pain, cognitive disturbances, difficulties in concentration, dizziness, headache, anxiety, depression, arthralgia, and a decline in quality of life (3, 4, 5, 6, 7, 8, 9).  Cellular damage, a robust innate immune response with the production of inflammatory cytokines, and a pro-coagulant state induced by SARS-CoV-2 infection may contribute to these sequelae. Survivors of previous coronavirus infections, including the SARS epidemic of 2003 and the Middle East Respiratory Syndrome (MERS) outbreak of 2012, exhibited Yesilar persistent symptoms, reinforcing concerns about clinically significant sequelae from COVID-19. Based on recent literature, long COVID is divided into two categories: (1) subacute or ongoing symptomatic COVID-19, which includes symptoms and abnormalities present from four to 12 weeks beyond acute COVID-19; and (2) chronic syndrome or post-COVID-19, which includes persistent symptoms and abnormalities present beyond 12 weeks of the onset of acute COVID-19 and not attributable to alternative diagnoses (10, 4, 5).  Many individuals experience persistent symptoms and a decline in quality of life after COVID-19 infection. Hospitalized individuals reported symptoms up to 110 days post-onset in the UK (3). |
| Objectives | 3 | State specific objectives, including any prespecified hypotheses | Pages 4-5 Clearly, this condition is a public health concern. There are varying definitions of long COVID, making it difficult to estimate its true prevalence worldwide. According to the World Health Organization (WHO), it is believed that at least 17 million people in the European Region experienced long COVID within the first two years of the pandemic. Data from the Centers for Disease Control and Prevention (CDC) regarding the U.S. population indicate that the prevalence of long COVID among non-institutionalized adults aged 18 and older decreased from 7.5% in June 2022 to 6.0% in June 2023, and from 18.9% to 11.0% among adults reporting a prior COVID-19 infection. After an initial decline, the prevalence has remained stable since 2023, with approximately one-quarter of adults with long COVID reporting significant activity limitations (11).  In Brazil, there are few studies that have assessed long COVID, making the objective of this investigation to evaluate the prevalence and associated factors of long COVID in the Brazilian population. |
| Methods | | |  |
| Study design | 4 | Present key elements of study design early in the paper | Page 5 Web survey study, observational, cross-sectional, and analytical with a quantitative approach (12). |
| Setting | 5 | Describe the setting, locations, and relevant dates, including periods of recruitment, exposure, follow-up, and data collection | Page 5 The reference population includes all Brazilians residing in Brazil at the time of the survey who had access to the questionnaire disseminated through social media (Facebook, Twitter, Instagram, WhatsApp, or email). Additionally, only participants aged 18 years or older who were diagnosed with COVID-19 between 2020 and 2023 were included. |
| Participants | 6 | (*a*) Give the eligibility criteria, and the sources and methods of selection of participants | Page 5-7 For participant recruitment, the Respondent-Driven Sampling (RDS) method adapted for online environments was used (12). Initially, data collection was conducted by selected researchers from all regions of Brazil in 2023. These researchers underwent four hours of pre-training to conduct an online survey. The RDS method used in this study was implemented as follows: a random selection of a set of participants (seeds) was made. The seeds were limited to ten referrals each in the first selection, and they managed this data in an Excel spreadsheet. The referral slots were limited to ensure enough candidates remained in the pool to continue the referral chain as much as possible within their networks. After the seeds made their referrals, each participant who returned contact via WhatsApp was interviewed and received Yesilar training to manage the spreadsheet with the ten referrals to be made. A new round of selection was generated when the referral spreadsheets were returned to the seeds. This back-and-forth replaced physical coupons and allowed data collection to be managed remotely.  Additionally, invitations to participate in the survey were disseminated through social media (Facebook, Twitter, Instagram, WhatsApp, or email). The data collection questionnaire was converted to a digital format. Invitations for participation in the survey were sent through messages on the aforementioned social media platforms, and the survey link was also posted. The link containing the questionnaire was made available on the researchers' social media profiles. It is emphasized that social media was used only as a means to publicize the research, ensuring that the collected information was solely that provided by the participant.  For the operationalization of data collection, participants received a link that included information about the nature and confidentiality of the research. By clicking on the link, participants were directed to the REDCap platform, where they accessed a question about participation in the study. Upon acceptance, participants were given access to the Informed Consent Form (ICF) and the study questionnaire.  The ICF for online data collection was available on the homepage, and participants could only access the questionnaire if they agreed to participate in the research by selecting the option “I have read and agree to participate in this research,” thereby giving their informed consent. In case of non-acceptance, the participant was directed to a closure page with thanks for their attention.  It is noteworthy that participants were guaranteed the right to have a second copy of the ICF, as it was made available for download, allowing them to complete it at their convenience. It is further highlighted that each Internet Protocol (IP) address could submit only one response. |
| Variables | 7 | Clearly define all outcomes, exposures, predictors, potential confounders, and effect modifiers. Give diagnostic criteria, if applicable | Page 7 The outcome variable chosen was “You had symptoms for four weeks or more” which is binary, allowing the calculation of odds and prevalence ratios. |
| Data sources/ measurement | 8* | For each variable of interest, give sources of data and details of methods of assessment (measurement). Describe comparability of assessment methods if there is more than one group | Page 7-8 The independent variables were social and demographic characteristics which selected variables were: Education (No education/Never completed any grade, Incomplete elementary education, Complete elementary education, Incomplete high school, Complete high school, Incomplete higher education, Complete higher education, Specialization, Masters, Doctorate, Postdoctoral), Sex (Female, Male), Confirmed diagnosis of COVID-19 (1 time, 2 times, 3 times, 4 times, 5 or more), Diagnosed with COVID-19 before vaccination (No, Yes), Chronic illness diagnosis before COVID-19 (No, Yes, I don’t know), Received antibiotics (No, Yes), Received prescribed antivirals (Yes, No), Received Ivermectin (No, Yes), Received chloroquine (No, Yes), Received home medicine (No, Yes), Received sedatives (No, Yes), Beneficiary of social programs (No, Yes), Income (Less than 1 minimum wage, 1 minimum wage, 2 minimum wages, 3 minimum wages, 4 minimum wages), Depression or Anxiety (Yes, No), Alcoholic drinks (No, Yes), Occupation (Unemployed, Employed, Self-Employed, Retired, Student).  The adapted questionnaire "COVID-19 Global Clinical Platform: Case Report Form for Post-COVID-19 Condition" was used in the study. This questionnaire was developed by the WHO to systematically collect information from patients about COVID-19 and contribute clinical data to the WHO Clinical Platform to expand knowledge on post-COVID-19 condition and support patient care and public health interventions. Additionally, it aims to gather standardized clinical data from individuals after hospital discharge or following acute illness to examine the medium- and long-term consequences of COVID-19. This questionnaire includes sociodemographic questions, as well as clinical questions related to COVID-19 and long COVID. |
| Bias | 9 | Describe any efforts to address potential sources of bias | Page 8 A pilot test was conducted where individuals were contacted through social media apps and invited to participate in the pilot study. Subsequently, 123 respondents were invited to provide feedback or comments about the survey via WhatsApp. All suggested changes were considered, along with minor adaptations in terminology. |
| Study size | 10 | Explain how the study size was arrived at | Page 6-7 For participant recruitment, the Respondent-Driven Sampling (RDS) method adapted for online environments was used (12). Initially, data collection was conducted by selected researchers from all regions of Brazil in 2023. These researchers underwent four hours of pre-training to conduct an online survey. The RDS method used in this study was implemented as follows: a random selection of a set of participants (seeds) was made. The seeds were limited to ten referrals each in the first selection, and they managed this data in an Excel spreadsheet. The referral slots were limited to ensure enough candidates remained in the pool to continue the referral chain as much as possible within their networks. After the seeds made their referrals, each participant who returned contact via WhatsApp was interviewed and received Yesilar training to manage the spreadsheet with the ten referrals to be made. A new round of selection was generated when the referral spreadsheets were returned to the seeds. This back-and-forth replaced physical coupons and allowed data collection to be managed remotely.  Additionally, invitations to participate in the survey were disseminated through social media (Facebook, Twitter, Instagram, WhatsApp, or email). The data collection questionnaire was converted to a digital format. Invitations for participation in the survey were sent through messages on the aforementioned social media platforms, and the survey link was also posted. The link containing the questionnaire was made available on the researchers' social media profiles. It is emphasized that social media was used only as a means to publicize the research, ensuring that the collected information was solely that provided by the participant.  For the operationalization of data collection, participants received a link that included information about the nature and confidentiality of the research. By clicking on the link, participants were directed to the REDCap platform, where they accessed a question about participation in the study. Upon acceptance, participants were given access to the Informed Consent Form (ICF) and the study questionnaire.  The ICF for online data collection was available on the homepage, and participants could only access the questionnaire if they agreed to participate in the research by selecting the option “I have read and agree to participate in this research,” thereby giving their informed consent. In case of non-acceptance, the participant was directed to a closure page with thanks for their attention. |
| Quantitative variables | 11 | Explain how quantitative variables were handled in the analyses. If applicable, describe which groupings were chosen and why | Page 9 Initially, the Fisher's exact test was conducted, and the outcome variable was crossed with the other qualitative variables included in the dataset. Subsequently, a logistic regression model was generated to estimate which of these variables provide evidence of being associated with the outcome of interest. |
| Statistical methods | 12 | (*a*) Describe all statistical methods, including those used to control for confounding | Page 8-9Initially, the Fisher's exact test was conducted, and the outcome variable was crossed with the other qualitative variables included in the dataset. Subsequently, a logistic regression model was generated to estimate which of these variables provide evidence of being associated with the outcome of interest.  To obtain evidence of the best model, the stepwise model selection method was considered, which automatically selects factors associated with an outcome within the context of a regression model. This method combines features of two other variable selection methods: forward selection, generally referred to as the forward method, and backward selection, commonly known as the backward method. |
|  |  | (*b*) Describe any methods used to examine subgroups and interactions | Page 9 To obtain evidence of the best model, the stepwise model selection method was considered, which automatically selects factors associated with an outcome within the context of a regression model. This method combines features of two other variable selection methods: forward selection, generally referred to as the forward method, and backward selection, commonly known as the backward method. |
|  |  | (*c*) Explain how missing data were addressed | Page 8 In total, 5,950 (five thousand nine hundred fifty) people were interviewed. However, with the aim of researching only those who responded to the symptom item over a period of four weeks or more in the General Health Questionnaire II, a filter was applied to the database, resulting in a final sample of 4,231 (four thousand two hundred thirty-one) people who effectively answered the question related to this variable. |
|  |  | (*d*) If applicable, describe analytical methods taking account of sampling strategy | Page 6 For participant recruitment, the Respondent-Driven Sampling (RDS) method adapted for online environments was used (12). Initially, data collection was conducted by selected researchers from all regions of Brazil in 2023. These researchers underwent four hours of pre-training to conduct an online survey. The RDS method used in this study was implemented as follows: a random selection of a set of participants (seeds) was made. The seeds were limited to ten referrals each in the first selection, and they managed this data in an Excel spreadsheet. The referral slots were limited to ensure enough candidates remained in the pool to continue the referral chain as much as possible within their networks. After the seeds made their referrals, each participant who returned contact via WhatsApp was interviewed and received Yesilar training to manage the spreadsheet with the ten referrals to be made. A new round of selection was generated when the referral spreadsheets were returned to the seeds. This back-and-forth replaced physical coupons and allowed data collection to be managed remotely.  Additionally, invitations to participate in the survey were disseminated through social media (Facebook, Twitter, Instagram, WhatsApp, or email). The data collection questionnaire was converted to a digital format. Invitations for participation in the survey were sent through messages on the aforementioned social media platforms, and the survey link was also posted. The link containing the questionnaire was made available on the researchers' social media profiles. It is emphasized that social media was used only as a means to publicize the research, ensuring that the collected information was solely that provided by the participant. |
|  |  | (*e*) Describe any sensitivity analyses |  |
| Results | | |  |
| Participants | 13* | (a) Report numbers of individuals at each stage of study—eg numbers potentially eligible, examined for eligibility, confirmed eligible, included in the study, completing follow-up, and analysed | Page 9 In total, 5,950 (five thousand nine hundred fifty) people were interviewed. However, with the aim of researching only those who responded to the symptom item over a period of four weeks or more in the General Health Questionnaire II, a filter was applied to the database, resulting in a final sample of 4,231 (four thousand two hundred thirty-one) people who effectively answered the question related to this variable |
|  |  | (b) Give reasons for non-participation at each stage | Page 9 However, with the aim of researching only those who responded to the symptom item over a period of four weeks or more in the General Health Questionnaire II, a filter was applied to the database, resulting in a final sample of 4,231 (four thousand two hundred thirty-one) people who effectively answered the question related to this variable |
|  |  | (c) Consider use of a flow diagram |  |
| Descriptive data | 14* | (a) Give characteristics of study participants (eg demographic, clinical, social) and information on exposures and potential confounders | Page 11-13 Answering “No” for “Have you had symptoms for a Period of four weeks or more” educational attainment varied, with 0.05% having no education or never completing any grade, 0.50% with incomplete elementary education, and 0.54% having completed elementary education. Additionally, 1.49% had incomplete high school, while 9.69% completed high school. Incomplete higher education was reported by 13.02%, and 8.79% completed higher education, with 6.15% holding a specialization. Advanced degrees were represented by 2.25% with a master's degree, 0.69% with a doctorate, and 0.17% at the postdoctoral level. In terms of sex, females comprised 28.93%, while males accounted for 3.36%. Regarding confirmed COVID-19 diagnoses, 31.41% had been diagnosed once, 9.88% twice, 1.82% three times, 0.31% four times, and 0.14% five or more times. A total of 20.35% were diagnosed with COVID-19 before vaccination, and 38.25% reported no prior chronic illness. Antibiotic use was reported by 21.59%, while 22.04% had received them. Among those who received prescribed antivirals, 32.42% indicated they had, and 29.04% did not receive Ivermectin. The majority, 40.07%, did not receive chloroquine, while 30.37% did not utilize home medicine. Sedatives were not taken by 41.36%, and 40.66% were not beneficiaries of social programs. Income distribution showed that 7.09% earned less than one minimum wage, 15.27% earned one minimum wage, and 7.85% earned two minimum wages. Regarding mental health, 12.70% reported having depression or anxiety, while 18.24% did not consume alcoholic drinks. In terms of occupation, 3.35% were unemployed, 20.90% were employed, 5.53% were self-employed, 1.57% were retired, and 12.19% were students.  With “Yes” for “Have you had symptoms for a period of four weeks or more” educational attainment included 0.14% with no education or never completing any grade, 1.02% with incomplete elementary education, and 0.85% having completed elementary education. Incomplete high school was reported by 1.73%, while 12.76% completed high school. A significant 17.30% had incomplete higher education, and 11.44% completed higher education. Specialization was held by 7.30%, with 2.93% possessing a master's degree, 0.66% with a doctorate, and 0.12% at the postdoctoral level. In terms of sex, females made up 26.59%, while males accounted for 8.11%. For confirmed COVID-19 diagnoses, 34.13% had been diagnosed once, 16.39% twice, 4.69% three times, 0.88% four times, and 0.36% five or more times. A significant 35.87% were diagnosed with COVID-19 before vaccination, and 45.48% reported having a prior chronic illness. Among participants, 19.43% had not received antibiotics, whereas 36.95% had. Regarding prescribed antivirals, 58.85% received them, and 33.90% reported having taken Ivermectin. Chloroquine was not received by 50.07%, and home medicine was utilized by 22.41%. Sedatives were taken by 5.11%, and 5.87% were beneficiaries of social programs. In terms of income, 9.62% earned less than one minimum wage, 22.31% earned one minimum wage, and 9.08% earned two minimum wages. Mental health issues were reported by 44.44%, and 34.67% consumed alcoholic drinks. In terms of occupation, 5.57% were unemployed, 25.58% were employed, 6.90% were self-employed, 1.50% were retired, and 16.92% were students.  The results indicate, at a 95% confidence level, that the following variables are associated with the outcome variable (p-value < 0.05): occupation, social program beneficiary status, income, number of COVID-19 diagnoses, COVID-19 diagnosis prior to vaccination, presence of chronic illness, depression or anxiety, receipt of antibiotics, receipt of prescribed antivirals, receipt of ivermectin, receipt of chloroquine, receipt of home remedies, and consumption of alcoholic beverages and sedatives.  A logistic regression model was then generated to estimate which of these variables provide evidence of being associated with the outcome of interest. Considering the association of these variables with the outcome variable. |
|  |  | (b) Indicate number of participants with missing data for each variable of interest | Page 9 However, with the aim of researching only those who responded to the symptom item over a period of four weeks or more in the General Health Questionnaire II, a filter was applied to the database, resulting in a final sample of 4,231 (four thousand two hundred thirty-one) people who effectively answered the question related to this variable |
| Outcome data | 15* | Report numbers of outcome events or summary measures | Pagr 11-13 The results indicate, at a 95% confidence level, that the following variables are associated with the outcome variable (p-value < 0.05): occupation, social program beneficiary status, income, number of COVID-19 diagnoses, COVID-19 diagnosis prior to vaccination, presence of chronic illness, depression or anxiety, receipt of antibiotics, receipt of prescribed antivirals, receipt of ivermectin, receipt of chloroquine, receipt of home remedies, and consumption of alcoholic beverages and sedatives. |
| Main results | 16 | (*a*) Give unadjusted estimates and, if applicable, confounder-adjusted estimates and their precision (eg, 95% confidence interval). Make clear which confounders were adjusted for and why they were included | Page 13-17A logistic regression model was then generated to estimate which of these variables provide evidence of being associated with the outcome of interest. Considering the association of these variables with the outcome variable.  It is observed, at a 95% confidence level, when analyzing the likelihood of experiencing COVID symptoms for 4 weeks or more, that a male individual has a 36.46% higher chance compared to a female (OR = 1.3646; CI = [1.1737; 1.5865]); those diagnosed with COVID prior to vaccination have a 22.30% increase compared to those who were not (OR = 1.2230; CI = [1.0587; 1.4126]); individuals using sedatives have a 24.50% increase compared to those who do not (OR = 1.2450; CI = [1.0782; 1.4378]); those consuming alcoholic beverages have a 34.95% increase compared to those who do not (OR = 1.3495; CI = [1.0272; 1.7569]); social program beneficiaries have a 47.29% increase compared to non-beneficiaries (OR = 1.4729); those with a chronic illness have a 33.47% increase compared to those without (OR = 1.3347; CI = [1.2004; 1.4866]); individuals who received antibiotics have a 39.48% increase compared to those who did not (OR = 1.3948; CI = [1.2025; 1.6176]); those who received antivirals have a 30.35% increase compared to those who did not (OR = 1.3035; CI = [1.1246; 1.5145]); a married individual has a 25.78% increase compared to a single individual (OR = 1.2578; CI = [1.0402; 1.5206]). Furthermore, an individual who has heard of the term has a 54.34% higher chance of experiencing COVID symptoms for 4 weeks or more compared to someone who has not (OR = 1.5434; CI = [1.3108; 1.8196]); those who sought healthcare services have a 67.28% increase compared to those who did not (OR = 1.6728; CI = [1.4484; 1.9328]); individuals hospitalized for symptoms have a 331.92% increase compared to those who were not (OR = 4.3192; CI = [2.5377; 7.8782]); a currently “employed” individual has a 23.62% decrease compared to an “unemployed” individual (OR = 0.7638; CI = [0.5861; 0.9920]); a “retired” individual has a 52.56% decrease compared to an “unemployed” individual (OR = 0.4744; CI = [0.2903; 0.7705]). The other occupational categories did not present statistical evidence of being related to the duration of COVID symptoms. An individual who received home remedies has a 30.38% increase in the likelihood of experiencing COVID symptoms for 4 weeks or more compared to someone who is “unemployed” (OR = 1.3038; CI = [1.1257; 1.5107]).  Once the interpretations have been made, the Receiver Operating Characteristic Curve (ROC) of the adjusted model is presented below. |
|  |  | (*b*) Report category boundaries when continuous variables were categorized | Page 10-13 Table 2 - Results of association tests between the outcome variable and the other variables considered for the study. Answering “No” for “Have you had symptoms for a Period of four weeks or more” educational attainment varied, with 0.05% having no education or never completing any grade, 0.50% with incomplete elementary education, and 0.54% having completed elementary education. Additionally, 1.49% had incomplete high school, while 9.69% completed high school. Incomplete higher education was reported by 13.02%, and 8.79% completed higher education, with 6.15% holding a specialization. Advanced degrees were represented by 2.25% with a master's degree, 0.69% with a doctorate, and 0.17% at the postdoctoral level. In terms of sex, females comprised 28.93%, while males accounted for 3.36%. Regarding confirmed COVID-19 diagnoses, 31.41% had been diagnosed once, 9.88% twice, 1.82% three times, 0.31% four times, and 0.14% five or more times. A total of 20.35% were diagnosed with COVID-19 before vaccination, and 38.25% reported no prior chronic illness. Antibiotic use was reported by 21.59%, while 22.04% had received them. Among those who received prescribed antivirals, 32.42% indicated they had, and 29.04% did not receive Ivermectin. The majority, 40.07%, did not receive chloroquine, while 30.37% did not utilize home medicine. Sedatives were not taken by 41.36%, and 40.66% were not beneficiaries of social programs. Income distribution showed that 7.09% earned less than one minimum wage, 15.27% earned one minimum wage, and 7.85% earned two minimum wages. Regarding mental health, 12.70% reported having depression or anxiety, while 18.24% did not consume alcoholic drinks. In terms of occupation, 3.35% were unemployed, 20.90% were employed, 5.53% were self-employed, 1.57% were retired, and 12.19% were students.  With “Yes” for “Have you had symptoms for a period of four weeks or more” educational attainment included 0.14% with no education or never completing any grade, 1.02% with incomplete elementary education, and 0.85% having completed elementary education. Incomplete high school was reported by 1.73%, while 12.76% completed high school. A significant 17.30% had incomplete higher education, and 11.44% completed higher education. Specialization was held by 7.30%, with 2.93% possessing a master's degree, 0.66% with a doctorate, and 0.12% at the postdoctoral level. In terms of sex, females made up 26.59%, while males accounted for 8.11%. For confirmed COVID-19 diagnoses, 34.13% had been diagnosed once, 16.39% twice, 4.69% three times, 0.88% four times, and 0.36% five or more times. A significant 35.87% were diagnosed with COVID-19 before vaccination, and 45.48% reported having a prior chronic illness. Among participants, 19.43% had not received antibiotics, whereas 36.95% had. Regarding prescribed antivirals, 58.85% received them, and 33.90% reported having taken Ivermectin. Chloroquine was not received by 50.07%, and home medicine was utilized by 22.41%. Sedatives were taken by 5.11%, and 5.87% were beneficiaries of social programs. In terms of income, 9.62% earned less than one minimum wage, 22.31% earned one minimum wage, and 9.08% earned two minimum wages. Mental health issues were reported by 44.44%, and 34.67% consumed alcoholic drinks. In terms of occupation, 5.57% were unemployed, 25.58% were employed, 6.90% were self-employed, 1.50% were retired, and 16.92% were students.  The results indicate, at a 95% confidence level, that the following variables are associated with the outcome variable (p-value < 0.05): occupation, social program beneficiary status, income, number of COVID-19 diagnoses, COVID-19 diagnosis prior to vaccination, presence of chronic illness, depression or anxiety, receipt of antibiotics, receipt of prescribed antivirals, receipt of ivermectin, receipt of chloroquine, receipt of home remedies, and consumption of alcoholic beverages and sedatives. |
|  |  | (*c*) If relevant, consider translating estimates of relative risk into absolute risk for a meaningful time period | N/A |
| Other analyses | 17 | Report other analyses done—eg analyses of subgroups and interactions, and sensitivity analyses | Page 17 Once the interpretations have been made, the Receiver Operating Characteristic Curve (ROC) of the adjusted model is presented below |
| Discussion | | |  |
| Key results | 18 | Summarise key results with reference to study objectives | Page 19-20 Several factors associated with an increased risk of long COVID have been identified, such as the use of sedatives, alcohol consumption, and comorbidities, as well as the positive role of vaccination against COVID-19. Furthermore, using association models like those presented in this study may help healthcare professionals identify patients at higher risk and adapt care more effectively.  The results of this study provide a detailed analysis of the interrelations between demographic, clinical, and behavioral factors with the persistence of COVID-19 symptoms for more than four weeks, highlighting the inherent complexity of the condition known as long COVID. Crucial factors for the exacerbation and prolongation of symptoms include the use of antibiotics and antivirals, prior hospitalization, unvaccinated individuals, consumers of alcohol and sedatives, as well as those with comorbidities and beneficiaries of social programs.  Conversely, it was observed that individuals in more stable occupational situations, as well as those who resort to traditional therapies, showed a lower probability of persistent symptoms, suggesting a direct relationship between socioeconomic factors and the clinical outcome of long COVID. The predictive robustness of the adjusted model, corroborated by the ROC curve analysis, underscores the need for more targeted preventive and therapeutic strategies, especially for vulnerable population groups. |
| Limitations | 19 | Discuss limitations of the study, taking into account sources of potential bias or imprecision. Discuss both direction and magnitude of any potential bias | Page 19 The limitations of this study includes selection bias due to reliance on a web-based survey that may underrepresent certain demographics, recall bias from self-reported data, and response bias in reporting behaviors like alcohol or sedative use. Additionally, the cross-sectional design limits causal inference, and potential confounding variables, such as mental health status, were not measured. These factors may affect the direction and magnitude of the observed associations, suggesting that the findings should be interpreted cautiously. Addressing these limitations in future research could improve the validity of conclusions regarding long COVID. |
| Interpretation | 20 | Give a cautious overall interpretation of results considering objectives, limitations, multiplicity of analyses, results from similar studies, and other relevant evidence | Page 20 This study offers valuable insights into the prevalence and risk factors of long COVID among the Brazilian population, revealing that male sex, prior COVID-19 infection before vaccination, and the use of sedatives and alcohol are associated with increased risks of prolonged symptoms. However, caution is warranted due to limitations such as potential selection and response biases from the web-based survey, reliance on self-reported data, and the cross-sectional design, which limits causal interpretations. Despite these challenges, the findings shows the importance of socioeconomic factors and emphasizes a need for targeted preventive and therapeutic strategies, particularly for vulnerable populations, and underscores the importance of an integrated, multidimensional approach to healthcare that helps professionals identify high-risk patients and adapt care effectively highlighting areas for future research to enhance understanding and healthcare strategies regarding long COVID. |
| Generalisability | 21 | Discuss the generalisability (external validity) of the study results | Page 19 This study emphasizes the need for targeted preventive and therapeutic strategies, particularly for vulnerable populations, and underscores the importance of an integrated, multidimensional approach to healthcare that helps professionals identify high-risk patients and adapt care effectively. |
| Other information | | |  |
| Funding | 22 | Give the source of funding and the role of the funders for the present study and, if applicable, for the original study on which the present article is based | Page 20 This study was financed in part by the Coordenação de Aperfeiçoamento de Pessoal de Nível Superior - Brasil (CAPES) - Finance Code 001 and CAPES - EPIDEMICS, Emergency Selection Notice IV CAPES, Impacts of the Pandemic. |

*Give information separately for exposed and unexposed groups.

**Note:** An Explanation and Elaboration article discusses each checklist item and gives methodological background and published examples of transparent reporting. The STROBE checklist is best used in conjunction with this article (freely available on the Web sites of PLoS Medicine at http://www.plosmedicine.org/, Annals of Internal Medicine at http://www.annals.org/, and Epidemiology at http://www.epidem.com/). Information on the STROBE Initiative is available at www.strobe-statement.org.
