# Supplementary material for: Prevalence and associated factors with long COVID in the Brazilian population: The role of health-related behaviors and sociodemographic characteristics
Source: PLoS One. 2026 Jan 2;21(1):e0339612. doi: 10.1371/journal.pone.0339612 (PMC12758746; doi:10.1371/journal.pone.0339612)
Supplement: Attachment — Submitted filename: Response to reviewers.docx [file pone.0339612.s006.docx]

Dear Editor,

We appreciate the opportunity to revise and resubmit our manuscript PONE-D-24-50830R1 entitled “Prevalence and associated factors with long COVID in the Brazilian population” and we thank you and Reviewer #2 for the valuable feedback provided in this round of review.

In the revised version, we have specifically addressed the reviewer’s observation regarding the Conclusion section. The beginning of the section was rewritten to ensure that it fulfills the role of a conclusion rather than discussion, making it more concise, focused, and aligned with the journal’s expectations. Interpretative content and detailed literature comparisons were removed from this section, and the text now succinctly summarizes the key findings, their implications, and recommendations for public health practice.

Additionally, we carefully reviewed the reference list to ensure that it is complete and accurate. Any cited papers that had been retracted were replaced with relevant and current references.

We believe this targeted revision improves the overall clarity, structure, and compliance of the manuscript with PLOS ONE’s editorial standards.

Below, we respond point by point to the reviewer’s comments.

Kind regards,
Martoreli Júnior et al.

**Reviewer #2:**

**6. Review Comments to the Author: The comments have been adequately addressed
The eerier parts of Conclusion section sounds like discussion- please consider rewriting it to sound like 'conclusion'**

**Answer:** We appreciate the reviewer’s observation. In order to avoid sounding like a discussion, we did not include methodological details or in-depth interpretations of the results. Instead, we summarized the findings objectively and prepared the reader for the following paragraphs, where specific factors and their implications are described. The first paragraph of the Conclusion has been thoroughly revised as below:

“This study provides a comprehensive overview of the factors associated with persistence of COVID 19 symptoms. Key findings indicate that demographic, clinical, and behavioral characteristics influence the likelihood of developing long COVID.”
