# Supplementary material for: Prevalence and associated factors with long COVID in the Brazilian population: The role of health-related behaviors and sociodemographic characteristics
Source: PLoS One. 2026 Jan 2;21(1):e0339612. doi: 10.1371/journal.pone.0339612 (PMC12758746; doi:10.1371/journal.pone.0339612)
Supplement: Attachment — Submitted filename: Reviewers report.docx [file pone.0339612.s005.docx]

**Reviewer’s report**:

**Title: Prevalence and associated factors with long COVID in the Brazilian population**

Comments to the Author

This manuscript involves a descriptive analysis of prevalence and associated factors with long COVID in Brazil. Data were obtained via an online survey using the WHO questionnaire, which incorporated signs and symptoms relating to mental health.

I have some comments that the authors might find helpful if revising the manuscript.

Abstract, you mentioned that “logistic regression model was adjusted”. In that case, you should present the adjusted odds ratio rather than the odds ratio in your results.

Methods, line 90: Replace “methdos” by “methods”

Methods, line 127: There is a missing comma here. Between “survey” and “The RDS”.

Methods, lines 173-174: “specially participants …. More?”: There seems to be repetition of essentially the same information with line 170. I suggest to reformulate or remove this sentence.

Methods, lines 184-185: please add a reference here.

Methods, line 212: Please add references for R software and Jamovi software

Methods, lines 204-205: I suggest to reformulate this sentence.

Methods, lines 204-205: More detail is required about the logistic regression model – e.g. how was missing data handled, how were the models generated ?

Methods, lines 210-211: Additionally, further information is needed regarding the construction of the ROC curve. For example, how was your dataset split?

Results, Table 2 and Table 3: The notation for the P values is very odd, please correct. Report exact p-values for all values greater than or equal to 0.001. P-values less than 0.001 may be expressed as p < 0.001 (see PLoS One statistical reporting guidelines <https://journals.plos.org/plosone/s/submission-guidelines.#loc-statistical-reporting>)

Results, lines 223-305: Generally there is too much information presented here and too much repetition from Table 3. Suggest highlighting two or three key results for each analysis in the text, but not reporting every result.

Results, line 274-276: This paragraph should be included in the 'Methods' section rather than the 'Results' section.

Results, line 281 : I have questions for Table 3. 1) If you are presenting the results of adjusting the logistic regression model then it should be made clear these are adjusted odds ratios. Please correct this in the table and throughout the documents. 2) This table is an output of the model results generated using the R software. It would be beneficial to rework the table for clearer presentation. 3) The presentation of the variable column should be reviewed. For example, for “sex” variable, what is the reference category for comparison? Is it male? Female? The same comment applies to all the other variables listed. The reference categories must be specified. 4) The 'Estimate' and 'Odds ratio' columns provide the same information. I suggest to remove one of them. 5) Numbers should be revised and presented in the English format.

Conclusion, line 313: Seems too long as a conclusion. Is it a conclusion or discussion section?

Conclusion, line 372: You present the result of the Hosmer-Lemeshow test here, despite not mentioning it in the Methods section. Why was this test used? Which question does it answer? Please explain this in the 'Statistical analysis' section of the Methods section.

Conclusion, line 396: You are interpreting your results as 'risk factors' when they are actually 'associated factors'. Review the manuscript and correct accordingly.

At last, it is really important to point out the relevance of the research work as it talks about the pprevalence and associated factors with long COVID which is already published in Brazil and by a number of other countries (see references below). Please give emphasis to this comment very well.

<https://www.scielo.br/j/csp/a/9zRf9w8hwnS9jShm5ysFgkC/?lang=en>

<https://www.mdpi.com/2227-9032/12/14/1443>

<https://www.cambridge.org/core/journals/infection-control-and-hospital-epidemiology/article/risk-factors-for-long-coronavirus-disease-2019-long-covid-among-healthcare-personnel-brazil-20202022/AA01F17E1C8A33C07457914E63AB3EEE>

<https://www.frontiersin.org/journals/medicine/articles/10.3389/fmed.2024.1344011/full>

<https://www.mdpi.com/2076-393X/12/1/99>

<https://bmcpublichealth.biomedcentral.com/articles/10.1186/s12889-025-22987-8>

<https://www.scielo.br/j/csp/a/9zRf9w8hwnS9jShm5ysFgkC/?lang=en>
