# Supplementary material for: Prevalence and associated factors with long COVID in the Brazilian population: The role of health-related behaviors and sociodemographic characteristics
Source: PLoS One. 2026 Jan 2;21(1):e0339612. doi: 10.1371/journal.pone.0339612 (PMC12758746; doi:10.1371/journal.pone.0339612)
Supplement: Attachment — Submitted filename: Response_to_reviewers_auresp_3.docx [file pone.0339612.s008.docx]

Dear Editor,

We appreciate the opportunity to revise and resubmit our manuscript “PONE-D-24-50830 – Prevalence and associated factors of long COVID in the Brazilian population.” We would also like to express our sincere gratitude to the Editor and Reviewers for their valuable comments and suggestions. In response to the feedback received, we made substantial revisions to improve the clarity, precision, and overall quality of the manuscript, including updating the title to better reflect reflect the study’s main independent variables.

The Results section was carefully revised to enhance clarity and readability, focusing on the most relevant findings while eliminating unnecessary repetition of information already presented in tables. Table formatting was standardized, and reference categories were clearly indicated for all variables. Sample size information was added to provide full transparency and allow readers to interpret the findings accurately. P-value reporting was adjusted to conform to journal standards, ensuring precise and consistent statistical presentation. The description of Fisher’s exact test was rewritten for clarity and accuracy. Table 3 was thoroughly reformatted to improve the readability and interpretability of the multivariate logistic regression results, highlighting reference categories, removing redundancies, and aligning the table with the methods described in the manuscript.

The Discussion section was expanded to contextualize the findings within the existing scientific literature and to highlight the implications for public health. Special attention was given to interpreting results in light of the sex imbalance in the study sample, emphasizing associated factors rather than implying causal risk factors. Terminology throughout the manuscript was standardized, grammatical inconsistencies were corrected, and long or complex sentences were simplified to improve readability. The Conclusion was condensed to focus exclusively on the key findings and their implications for public health, avoiding repetition of information already addressed in the Discussion.

In addition, the overall manuscript structure was revised to improve logical flow and coherence, with the Discussion now appropriately preceding the Conclusion.

We greatly appreciate the opportunity to revise our manuscript and believe that these modifications have substantially improved its quality, readability, and scientific contribution.

Below, we respond point by point to the comments of the Academic Editor and Reviewers.

Kind regards,
Martoreli Júnior et al.

**ACADEMIC EDITOR:**

**Abstract, you mentioned that “logistic regression model was adjusted”. In that case, you should present the adjusted odds ratio rather than the odds ratio in your results.**

**Answer:** The phrase was rewritten for better understanding and the adjusted odds ratio was presented.

**Methods, line 90: Replace “methdos” by “methods”**

**Answer:** Replaced

**Methods, line 127: There is a missing comma here. Between “survey” and “The RDS”.**

**Answer:** Comma was added.

**Methods, lines 173-174: “specially participants …. More?”: There seems to be repetition of essentially the same information with line 170. I suggest to reformulate or remove this sentence.**

**Answer:** The sentence was removed to avoid redundancy, as the information was already presented in line 170. The paragraph was reformulated to maintain clarity without repeating content.

**Methods, lines 184-185: please add a reference here.**

**Answer:** A reference was added to support the statement.

**Methods, line 212: Please add references for R software and Jamovi software**

**Answer:** References for both R software (R Core Team) and Jamovi (The Jamovi Project) were inserted.

**Methods, lines 204-205: I suggest to reformulate this sentence.**

**Answer:** The sentence was reformulated.

**Methods, lines 204-205: More detail is required about the logistic regression model – e.g. how was missing data handled, how were the models generated ?**

**Answer:** For variables with at least one cell in the columns or rows containing a value of zero, the test could not be performed, and therefore they were not included in the model. This statement was included in the “Statistical analysis” section.

**Methods, lines 210-211: Additionally, further information is needed regarding the construction of the ROC curve. For example, how was your dataset split?**

The ROC curve was generated using the predicted probabilities obtained from the final logistic regression model fitted to the entire dataset, without performing a train/test split. We acknowledge that using the same dataset for model development and performance assessment may lead to optimistic estimates of predictive accuracy. Therefore, the ROC analysis should be interpreted considering this limitation.

**Results, Table 2 and Table 3: The notation for the P values is very odd, please correct. Report exact p-values for all values greater than or equal to 0.001. P-values less than 0.001 may be expressed as p < 0.001 (see PLoS One statistical reporting guidelines** [**https://journals.plos.org/plosone/s/submission-guidelines.#loc-statistical-reporting**](https://journals.plos.org/plosone/s/submission-guidelines.#loc-statistical-reporting)**)**

**Answer:**

**Results, lines 223-305: Generally there is too much information presented here and too much repetition from Table 3. Suggest highlighting two or three key results for each analysis in the text, but not reporting every result.**

**Answer:**

**Results, line 274-276: This paragraph should be included in the 'Methods' section rather than the 'Results' section.**

**Answer:**

**Results, line 281 : I have questions for Table 3. 1) If you are presenting the results of adjusting the logistic regression model then it should be made clear these are adjusted odds ratios. Please correct this in the table and throughout the documents. 2) This table is an output of the model results generated using the R software. It would be beneficial to rework the table for clearer presentation. 3) The presentation of the variable column should be reviewed. For example, for “sex” variable, what is the reference category for comparison? Is it male? Female? The same comment applies to all the other variables listed. The reference categories must be specified. 4) The 'Estimate' and 'Odds ratio' columns provide the same information. I suggest to remove one of them. 5) Numbers should be revised and presented in the English format.**

**Answer:** The entire table was revised. All reference category variables were included in the table.

**Conclusion, line 313: Seems too long as a conclusion. Is it a conclusion or discussion section?**

**Answer:** We have added the Discussion section, which is now followed by the Conclusion, as pointed.

**Conclusion, line 372: You present the result of the Hosmer-Lemeshow test here, despite not mentioning it in the Methods section. Why was this test used? Which question does it answer? Please explain this in the 'Statistical analysis' section of the Methods section.**

Thank you for your observation. The Hosmer–Lemeshow test was used to assess the calibration of the logistic regression model, that is, the extent to which the predicted probabilities agree with the observed outcomes across different risk groups. This test was therefore included to evaluate whether the model was appropriately fitted to the study data. We have now added an explicit description of its use and purpose in the ‘Statistical analysis’ section of the Methods.

The authors add in the methods sections; “In addition to evaluating the discriminative ability of the model using the ROC curve and AUC, the calibration of the logistic regression model was assessed using the Hosmer–Lemeshow goodness-of-fit test. This test verifies whether the predicted probabilities of the outcome adequately reflect the observed frequencies across deciles of risk. A non-significant p-value indicates that the model is well calibrated, whereas a significant result suggests poor fit. Although the ROC curve assesses the model’s ability to discriminate between individuals with and without the outcome, the Hosmer–Lemeshow test answers a different question, namely whether the estimated probabilities are accurate when compared to the real data distribution.”

**Conclusion, line 396: You are interpreting your results as 'risk factors' when they are actually 'associated factors'. Review the manuscript and correct accordingly.**

**The term were carefully review through the entire manuscript and changed.**

**At last, it is really important to point out the relevance of the research work as it talks about the pprevalence and associated factors with long COVID which is already published in Brazil and by a number of other countries (see references below). Please give emphasis to this comment very well.**

<https://www.scielo.br/j/csp/a/9zRf9w8hwnS9jShm5ysFgkC/?lang=en>

<https://www.mdpi.com/2227-9032/12/14/1443>

<https://www.cambridge.org/core/journals/infection-control-and-hospital-epidemiology/article/risk-factors-for-long-coronavirus-disease-2019-long-covid-among-healthcare-personnel-brazil-20202022/AA01F17E1C8A33C07457914E63AB3EEE>

<https://www.frontiersin.org/journals/medicine/articles/10.3389/fmed.2024.1344011/full>

<https://www.mdpi.com/2076-393X/12/1/99>

<https://bmcpublichealth.biomedcentral.com/articles/10.1186/s12889-025-22987-8>

<https://www.scielo.br/j/csp/a/9zRf9w8hwnS9jShm5ysFgkC/?lang=en>

**Answer:** We are immensely grateful for your highlighting of the relevance and importance of our work. We fully agree that it is crucial to emphasize the pertinence of our research in relation to long COVID, an ongoing and widely recognized global public health challenge, with a growing body of literature, including studies in Brazil, as the Reviewer pointed out. In response to your observation, we have revised our Conclusion section to ensure that the relevance of the study is communicated with the necessary emphasis. We have inserted a new paragraph at the very beginning of this section to position our work within the context of the existing literature and reiterate how our detailed analysis of risk factors in a Brazilian population is a timely and vital contribution to health policy planning and monitoring the post-COVID-19 situation in the country. We believe this modification reinforces the impact of our study.

**ACADEMIC EDITOR:**

Changes required for acceptance:

**Formatting and Language:**

**- Review the entire manuscript for grammar, punctuation, and stylistic consistency, simplifying unnecessarily long or complex sentences.**

**Answer:** We carefully revised the entire manuscript to correct grammar, punctuation, and stylistic inconsistencies. Additionally, unnecessarily long or complex sentences were simplified to improve clarity and readability.

**- The manuscript has improved substantially but remains somewhat verbose, with occasional repetition of results. Please revise the text for conciseness, especially in the Results section.**

**Answer:** We further edited the manuscript to improve conciseness, particularly in the Results section, by removing repetitive descriptions and focusing on the key findings.

**- Standardize formatting for all headings and tables according to journal style (e.g., avoid all-caps titles).**

**Answer:** All headings and tables were reformatted to comply with the journal's style guidelines, including replacement of all-caps titles with appropriate capitalization.

**- Ensure consistent terminology throughout (e.g., use associated factors rather than risk factors).**

We revised the manuscript to ensure terminology consistency and replaced all instances of “risk factors” with “associated factors”.

**Title: Revise the title to better reflect the study’s main independent variables, emphasizing the assessment of healthy habits and sociodemographic characteristics.**

The title has been updated to better reflect the study’s primary independent variables, specifically highlighting healthy habits and sociodemographic characteristics. Prevalence and associated factors with long COVID in the Brazilian population: The role of health-related behaviors and sociodemographic characteristics.

**Abstract:**

**- Simplify the abstract for greater clarity and brevity, avoiding unnecessary methodological details (e.g., mention of basic descriptive analyses).**

The abstract was rewritten to improve clarity and brevity by removing nonessential methodological details.

**- When presenting results from multivariate models, report adjusted odds ratios rather than crude values.**

Adjusted odds ratios are now reported in place of crude values in both the abstract and Results section.

**- Include a concise statement identifying the knowledge gap that motivated the study, supported by references that contextualize the importance of investigating long COVID in Brazil.**

A concise statement identifying the knowledge gap was added to the abstract and introduction, supported by appropriate references: “Despite increasing global attention to long COVID, evidence from Latin American countries remains limited. Brazil presents unique epidemiological, social, and healthcare characteristics that may influence long COVID outcomes, highlighting the need for context-specific data. Recent studies conducted in Brazil and across Latin America demonstrate substantial prevalence and long-term impact of the condition » **Azambuja P, Bastos LSL, Batista-da-Silva AA, Ramos GV, Kurtz P, Dias CMC, et al. Prevalence, risk factors, and impact of long COVID in a socially vulnerable community in Brazil: a prospective cohort study. *Lancet Reg Am.* 2024;37:100839. doi: 10.1016/j.lana.2024.100839.**

**Salci MA, Carreira L, Oliveira NN, Pereira ND, Covre ER, Pesce GB, et al. Long COVID among Brazilian Adults and Elders 12 Months after Hospital Discharge: A Population-Based Cohort Study. *Healthcare (Basel).* 2024;12(14):1443. doi: 10.3390/healthcare12141443.**

**Angarita-Fonseca A, Torres-Castro R, Benavides-Cordoba V, Chero S, Morales-Satán M, Hernández-López B, et al. Exploring long COVID condition in Latin America: Its impact on patients’ activities and associated healthcare use. *Front Med (Lausanne).* 2023;10:1168628. doi: 10.3389/fmed.2023.1168628.**

**Introduction: Expand the contextual background to highlight the scarcity of national evidence on long COVID and the relevance of this study for public health planning.**

**Answer:** We expanded the introduction to emphasize the lack of national evidence on long COVID and strengthen the public health relevance of this study.

**Materials and Methods:**

**- Correct minor typographical and grammatical issues (e.g., “methdos” → “methods”) and improve sentence structure in sections describing ethical aspects and data collection procedures.**

**Answer:** All typographical and grammatical errors were corrected, and sentence structure was improved in the sections describing ethical procedures and data collection.

**- Provide additional information about the logistic regression model, including handling of missing data, model generation, and ROC curve construction.**

**Answer:** Additional details regarding model development, missing data management, and ROC curve construction were added to the Methods section.

**- Add appropriate references for the questionnaire used and its prior validation or adaptation.**

The questionnaire used as a base is Global COVID-19 Clinical Platform Case Report Form (CRF) for Post COVID condition (Post COVID-19 CRF) them in the Study design and setting was describe and referenced and the additional scales that were incorporated into the adapted questionnaire were now mentioned in the same paragraph. Furthermore we added that the full questionnaire can be provided upon request to the authors.

**- Include references for the statistical software (R and Jamovi).**

**Anwer:** References for R and Jamovi software have been added.

**- Explicitly incorporate the data availability statement into the main text for transparency and compliance.**

**Anwer:** The data availability statement was incorporated directly into the manuscript.

**Results:**

**- Reduce redundancy between the text and tables, focusing on key findings rather than restating all numerical results.**

**Anwer:** The Results section was revised to avoid redundancy with tables, highlighting only essential findings.

**- Remove the written-out numbers in parentheses (e.g., “5,950 (five thousand nine hundred fifty)”).**

**Anwer:** All instances of written-out numerical values in parentheses were removed.

**- Shorten overly long sentences for clarity and verify that table references are consistent.**

**Anwer:** Overly long sentences were shortened, and all table references were checked and corrected where necessary.

**- Clearly indicate reference categories for all variables in Table 3.**

**Anwer:** The entire table was revised. All reference category variables were included in the table.

**- Revise Table 3 for clearer presentation — remove redundant columns, use standard English numeric formatting, and report exact p-values following journal statistical reporting guidelines.**

**Anwer:** Table 3 was reformatted to remove redundant columns, apply standard English numerical formatting, and include exact p-values in line with reporting guidelines.

**- Provide the sample size and complete data for each variable, ensuring all categories of education and occupation are clearly described.**

**Anwer:** Sample sizes and full data descriptions, including detailed education and occupation categories, were added in the firs paragraph of the Results.

**- Relocate the description of the ROC curve to the Methods section.**

**Anwer:** The description of the ROC curve was moved from the Results to the Methods section.

**Discussion and Conclusion:**

**- Reorganize sections so that the Discussion precedes the Conclusion.**

**Anwer:** The manuscript structure was revised so that the Discussion section now precedes the Conclusion.

**- In the Discussion, acknowledge the unequal gender distribution and interpret sex-related findings with caution.**

**Anwer:** However, these findings must be interpreted with caution due to the unequal gender distribution in the sample, which may have introduced bias and limited the robustness of sex-related comparisons.

**- Standardize the reporting of odds ratios and confidence intervals (e.g., OR = 1.39, CI = 1.20–1.61).**

**Anwer:** All odds ratios and confidence intervals were reformatted according to the recommended style.

**- Revise ambiguous or grammatically inconsistent phrases to improve readability.**

**Anwer:** Ambiguous and grammatically inconsistent phrases were revised throughout the manuscript to enhance readability.

**- Justify the inclusion of the Hosmer–Lemeshow test within the Methods section.**

**Anwer:** Thank you for your observation. The Hosmer–Lemeshow test was used to assess the calibration of the logistic regression model, that is, the extent to which the predicted probabilities agree with the observed outcomes across different risk groups. This test was therefore included to evaluate whether the model was appropriately fitted to the study data. We have now added an explicit description of its use and purpose in the ‘Statistical analysis’ section of the Methods.

In addition to evaluating the discriminative ability of the model using the ROC curve and AUC, the calibration of the logistic regression model was assessed using the Hosmer–Lemeshow goodness-of-fit test. This test verifies whether the predicted probabilities of the outcome adequately reflect the observed frequencies across deciles of risk. A non-significant p-value indicates that the model is well calibrated, whereas a significant result suggests poor fit. Although the ROC curve assesses the model’s ability to discriminate between individuals with and without the outcome, the Hosmer–Lemeshow test answers a different question, namely whether the estimated probabilities are accurate when compared to the real data distribution.

**- Condense the Conclusion to focus on the principal findings and public health implications, avoiding repetition of points already discussed.

Anwer:** The Conclusion section was condensed to focus solely on the main findings and their implications for public health. Repetitive content previously addressed in the Discussion was removed.

**Reviewer #3: This revised manuscript presents a cross-sectional web survey detailing the prevalence and associated factors of long COVID in the Brazilian population. The authors have adequately addressed the previous reviewer comment regarding the conclusion section, revising it to summarize the key findings rather than presenting an extended discussion.**

**The manuscript is now technically sound and clearly written, the statistical methods (Fisher's exact test and stepwise logistic regression) are appropriate for the data structure and objectives, however, the response to the data availability requirements, while present in the submission forms, needs to be explicitly integrated into the main manuscript text for clarity and compliance.**

**Anwer:** Thank you for the constructive comments. The data availability statement has now been explicitly incorporated into the main manuscript text to ensure full compliance.

**Reviewer #4: The authors conducted a cross-sectional study using the web to examine the prevalence and associated factors of long COVID in the Brazilian population. They found that factors like sedative and alcohol use were linked to a higher risk of long COVID, while vaccination showed a positive impact.**

**Overall, if the research questions examined in this study were to be replicated in numerous robust studies, the findings could be significant for implementing public health interventions during pandemics like COVID-19.

With that in mind, this reviewer has the following to remark:**

**1. Abstract
The abstract serves as a concise summary of the study. The text needs editing to make it more concise and improve readability. For instance, there is no need to state here, “Descriptive statistics were performed…” It’s a given that the authors start their statisical analysis with a look at descriptive statistics first, then move on to bivariate and multivariate analyses.**
**Anwer:** The abstract has been revised for conciseness and improved readability, and unnecessary methodological descriptions, including the mention of descriptive statistics, have been removed.

**2. Matrials and Methods
Under Ethical Aspects (lines 95-96), the authors write, “Regarding confidentiality and privacy, it is emphasized that these is maintained. The information is confidential, and participants is not identified at any time.”

These sentences should be edited to improve their grammatical accuracy and readability.**
**Anwer:** The sentence was corrected for grammatical accuracy and clarity.

**3. Sample and data collection procedures
The authors state (lines 126-129), “In total 39 of these researchers underwent four hours of pre-training to conduct an online survey The RDS method used in this study was implemented as follows: a random selection of a set of participants (seeds) was made.”

Is this one sentence or two? If two, punctuation is missing.

Anwer:** The sentence was edited to separate it correctly into two sentences and improve readability.

**4. Study variables
4.1 (Lines 169-185)
One issue here is the outcome variable, specifically the four-week criterion used by the authors as a marker for ‘Long COVID.’

If there is a way to align the definition of ‘Long COVID’ with current guidelines, including the Delphi-approach consensus, it should be pursued because it would strengthen the study.

In this context, I wonder if the participants provided more details that could help the authors refine their study from the data collected about their outcome variable. For example, after asking participants, “Did you have symptoms for four weeks or more?” were there follow-up questions like, “For how long have you had symptoms?”**

**Anwer:** We acknowledge this important point. Where possible, we aligned the definition of long COVID with current international guidelines, including Delphi consensus recommendations. A clarification was added previously, and study limitations were noted regarding the four-week criterion.
For the follow-up questions such as “For how long have you had symptoms?” unfortunately at the time it was not included in the survey.

**4.2 (Lines 186-187)
The authors state, “The independent variables were social and demographic characteristics which selected variables were: Education…”

This is another sentence that needs editing for smoother reading.**
**Anwer:** The sentence was revised to improve clarity and flow.

**5. Results
5.1 (Line 214)
The title is in all caps, which doesn’t follow the overall layout of the paper.**
**Anwer:** The title was reformatted to align with the journal’s formatting standards.

**5.2 (Line 215)
The authors state, “In total, 5,950 (five thousand nine hundred fifty) people were interviewed.”**

**Adding the text in parentheses is unnecessary; some readers may find it redundant.**

**Anwer:** All written-out numbers in parentheses were removed.
**5.3 (Line 218)
The authors write, “...4,231 (four thousand two hundred thirty-one)…”
Again, there is no need to add the text in parentheses.
Anwer:** All written-out numbers in parentheses were removed.

**5.4 (Lines 223-226)
The authors state, “Fisher's exact test was subsequently performed with the dichotomous variable "Have you had symptoms for a period of 4 weeks or more?" and the other variables in the form, presenting p-value < 0.05 with the variables in Table 2, note that some participants did not respond to all questions presented in this tables, consequently, the total count for each variable may vary.”

This sentence is too long and confusing. Do the authors mean Table 2? If so, why do they say “this tables” in line 226?**

**It would be advisable to edit this sentence. Make it at least two sentences. The first one could end with “Table 2,” a complete sentence. It would seem best to create a new sentence for what follows: “Note that some…”**
**Anwer:** The sentence was divided into shorter sentences and corrected to refer specifically to “Table 2”.

**5.5 (Lines 230-235)
This paragraph could benefit from editing and paraphrasing:

Answering “No” for “Have you had symptoms for a period of four weeks or more,” educational attainment varied, with 0.17% having no education or never completing any grade, 1.03% with incomplete elementary education, and 1.20% having completed elementary education. Additionally, 3.26% had incomplete high school, while 21.61% completed high school.
Incomplete higher education was reported by 29.33%, and 19.38% completed higher education, with 13.03% holding a specialization.**

**Anwer:** The paragraph was rewritten to improve clarity and conciseness.

**5.6 (Lines 237-238)
The authors state, “In terms of sex, females comprised 89.88%, while males accounted for 10.12%.”

This clearly indicates a skewed sample, with females being more interested in this survey than males.**
**Anwer:** We agree and added clarification regarding the skewed sex distribution and the need for cautious interpretation in the Discussion section.

**5.7 (Lines 241-242)
The authors state, “Antibiotic use was reported by 55.23%, while 44.77% had not received them.”

The second portion of this sentence is unnecessary and can be deleted.**
**Anwer:** Paragraph rewritten.

**5.8 (Lines 278-279)
The authors write, “For each variable included in the final model, the specific response category associated with the outcome..”

Double punctuation?**

**Anwer:** The punctuation was corrected.
5.9 (Lines 283-285)
**The authors write, “It is observed, at a 95% confidence level, when analyzing the likelihood of experiencing COVID symptoms for 4 weeks or more, that a male individual has a 36.46% higher chance compared to a female (OR = 1.36 CI= 1.17-1.58);”

As mentioned in comment 5.6, we are dealing with a skewed sample, with males making up just over 10%. Therefore, any conclusions drawn about males versus females in this analysis should be taken with caution. I suggest the authors elaborate on this in their discussion section.**

**Anwer:** This concern was addressed in the Discussion, and we now explicitly acknowledge that sex-related findings should be interpreted cautiously due to sample imbalance.

**5.10 (Lines 295-297)
The authors write, “Furthermore, an individual who has heard of the term has a 54.34% higher chance of experiencing COVID symptoms for 4 weeks or more compared to someone who has not (OR = 1.54 CI= 1.31-1.81);

To clarify, the authors should specify here which term they are referring to. Therefore, the sentence could be rewritten as: “Furthermore, an individual who has heard of the term ‘Long COVID’ has a 54.34% higher chance of experiencing COVID symptoms for 4 weeks or more compared to someone who has not (OR = 1.54, CI = 1.31-1.81);”**

**Anwer:** Paragraph rewritten.

**6. Discussion/conclusion
6.1 (Line 313)
It is advisable to place the conclusion section after the discussion section, rather than the other way around.
Anwer:** The manuscript structure was modified accordingly.

**6.2 (Lines 317-318)
The authors write, “It was observed that men have a 36.46% higher chance of developing what is termed long COVID compared to women (OR = 1.36 CI= 1.17-1.58).”

Please refer to my comments 5.6 and 5.9.
Anwer:** The Discussion section now clearly reflects the caution regarding interpretation of sex-related results, as recommended.

**6.3 (Lines 345-346)
The authors write, “The use of antibiotics (OR = 1.3948 CI= 1.20-1.61) and antivirals (OR = 1.3035 CI= 1.12-1.51) was also associated with a higher risk of persistent symptoms.”

The reported odds ratios should follow the same style as confidence intervals. So the sentence could be edited to:“The use of antibiotics (OR = 1.39 CI= 1.20-1.61) and antivirals (OR = 1.30 CI= 1.12-1.51) was also associated with a higher risk of persistent symptoms.”**

**Anwer:** The sentence was edited as suggested, standardizing the format.

6.4 (Lines 351-354)
**The authors state, “Additionally, the use of home remedies was associated with a 30.38% increase in the risk of prolonged symptoms (OR = 1.30 CI= 1.12-1.51), suggesting that these treatments may, in some cases could not be effective.”**

**This sentence needs attention.**

**Anwer:** The sentence was revised for grammatical correctness and clarity.

**6.5 (Lines 357-358)
The authors write, “Individuals who were hospitalized for COVID-19 presented a 331.92% increase in the probability of persistent symptoms (OR = 4.3192 CI= 2.53-7.87)…”

As mentioned in comment 6.3, the reported odds ratios should follow the same style as confidence intervals, which means that the above sentence could be edited to “Individuals who were hospitalized for COVID-19 presented a 331.92% increase in the probability of persistent symptoms (OR = 4.32 CI= 2.53-7.87)…”**

**Anwer:** The sentence was corrected.

**6.6 (Lines 384-406)
These lines could be presented as a conclusion section. In other words, line 313 should become “Discussion.”**

**Anwer:** These adjustments were implemented as suggested.

Reviewer #5: (No Response)

**Reviewer #6: Title: The manuscript's title addresses general factors associated with long COVID. However, the methods section describes a set of characteristics or independent variables, specifically healthy habits and sociodemographic characteristics. Therefore, I recommend modifying the title to align with these independent variables.
Anwer:** Title modified.
**Abstract: The authors state in line 86 on page 4, "In Brazil, there are few studies that have assessed long COVID, making the objective of this investigation to evaluate the prevalence and associated factors of long COVID in the Brazilian population." However, the absence of references and a clear gap in knowledge regarding long COVID is not evident.**

**Anwer:** The manuscript was reviewed to ensure this gap.

**Sample and data collection procedures: While sampling was described, the sample size was not provided. Furthermore, details regarding the pilot study and the properties of the questionnaire are missing, as are references or previous data that utilized those questionnaires**.

**Anwer:** In the Results we’ve rewritten the first line to make more clear : A total of 4,231 individuals were included in the final sample.

The Study design and setting section now details the origin, adaptations, and references of the questionnaire used, including the modifications implemented for this study. Additionally, we added a statement informing that the full questionnaire can be provided upon request to the authors. Information regarding the pilot study, including its procedures and objectives, is described in the Data source section.

**Results: Please provide the missing data for each variable. Additionally, review and clarify the number of categories for education and occupation.**

**Answer:** Education was categorized into 11 levels, ranging from no formal education to postdoctoral degree. Occupation was classified into five categories (unemployed, employed, self-employed, retired, student). In the multivariate logistic regression model, each category of these variables was individually tested against the reference category (as described in the Statistical Methods section), allowing the estimation of the adjusted effect of each level on the likelihood of long COVID.

For variables with at least one cell in the columns or rows containing a value of zero, the test could not be performed, and therefore they were not included in the model.

To address this, the entire descriptive section was revised and all ambiguous elements were removed.

**Table 3: Language Questions for Review.**

**Answer:** Table 3 was reviewed and revised for linguistic clarity, formatting consistency, and adherence to journal statistical guidelines.
