# Supplementary material for: Prevalence and associated factors with long COVID in the Brazilian population: The role of health-related behaviors and sociodemographic characteristics
Source: PLoS One. 2026 Jan 2;21(1):e0339612. doi: 10.1371/journal.pone.0339612 (PMC12758746; doi:10.1371/journal.pone.0339612)
Supplement: Attachment — Submitted filename: Reviewer 3 report.docx [file pone.0339612.s007.docx]

**Reviewer’s report**:

**Title: Prevalence and associated factors with long COVID in the Brazilian population: The role of the health-related behaviors and sociodemographic characteristics**

Comments to the Author

This manuscript involves a descriptive analysis of prevalence and associated factors with long COVID in Brazil. Data were obtained via an online survey using the WHO questionnaire, which incorporated signs and symptoms relating to mental health.

Thank you for taking the previous comments into consideration.

Please find below some minor comments:

In the abstract : Lines 36-44 : Pay attention to the separator between the OR and CI values. It should be a full stop « . » instead of a comma « , ».

Lines 262-263 : Table 1, Starting from column 3, replace the commas « , » by full stops « . » for the numbers provided.

Line 273-274 : Table 2. In the column on p-values. For the p-value values, replace « , » by « . »

Line 343 : Table 3, same comment. Replace « , » by « . »
